# “I only smoke when I have nothing to do”: a qualitative study on how smoking is part of everyday life in a Greenlandic village

**DOI:** 10.3402/ijch.v72i0.21657

**Published:** 2013-08-05

**Authors:** Anne Birgitte Jensen, Lise Hounsgaard

**Affiliations:** 1 Queen Ingrid's Hospital, Nuuk, Greenland; 2 Institute of Nursing and Health Sciences, University of Greenland, Nuuk, Greenland; 3 Research Unit of Nursing, Institute of Clinical Research, University of Southern Denmark, Odense, Denmark

**Keywords:** ethnographic design, Greenland, self-stigmatisation, smoking behavior, smoking cessation, withdrawal symptoms

## Abstract

**Background:**

Smoking-related illnesses, such as chronic obstructive pulmonary disease, cardiovascular disease and lung cancer, are common in Greenland. Factors such as age, gender, cigarette use, restricted smoking at home and socio-economic determinants are well-known predictors for smoking and smoking cessation. In 2005, 66% of the adult population in were Greenland smokers, despite widespread smoking cessation campaigns. It is therefore imperative to identify the factors that influence the low levels of smoking cessation to be able to offer cessation interventions of high quality.

**Aim:**

To develop knowledge about how smoking forms an incorporated part of a social and cultural context in the daily lives of unskilled residents of a small town in northern Greenland.

**Design:**

An ethnographic field study was carried out in 2010, including participant observation, informal conversation with health professionals and semi-structured interviews with 4 smokers (2 women and 2 men). Data were analysed with a phenomenological hermeneutic approach.

**Results:**

All informants were daily smokers. During work hours, they smoked fewer cigarettes due to control policy as well as having something to do. At home, they smoke more during leisure time. Having time on one's hands can be a factor in smokers remaining as smokers. It appears that smokers seem to consider themselves to be stigmatised. This may be one reason for wanting to stop smoking. Smokers ask how to quit and also ask for help to give up smoking with regard to medical treatment for withdrawal symptoms. Serious illness and pregnancy both appear to be triggers to consider giving up smoking. Severe withdrawal symptoms and lack of knowledge about how to give up smoking are barriers to participants achieving their goal.

**Conclusion:**

Prevention initiatives should be targeted at all smokers and a smoking cessation service should be developed, where smokers are supervised and receive medical treatment for withdrawal symptoms.

The number of restrictions on smoking in public and private spaces in Greenland is rising. Despite various smoking cessation campaigns, preventative initiatives including radio campaigns in 2010 and smoking legislation which restricted smoking in public places, 66% of the adult population in Greenland were smokers in 2005. It is therefore imperative to identify the factors that influence the low levels of smoking cessation to be able to offer cessation interventions of high quality. To be able to understand smoking from “inside” in its social and cultural context, an ethnographic field study was carried out to develop knowledge about how smoking is incorporated in daily living.

Smoking-related illnesses such as chronic obstructive pulmonary disease and cardiovascular disease are very prevalent in Greenland. Lung cancer is the most common form of cancer, with double the incidence rates of Denmark (1). In Greenland in 2005–2007, the proportion of adult smokers over the age of 15 was 66% (2), compared to 25% in Denmark (3). The issue of smoking has priority in the government's public health programme (Inuuneritta), and the stated goal is that, at the end of 2012, only 40% of the adult population will be smokers (4).

Between 1999 and 2005, a downward trend in numbers of smokers can be seen compared to 1993, with fewer smokers in all social groups. It is, however, the unemployed and unskilled workers who make up the largest group of smokers (70–80%) (2). More men than women give up smoking. A population survey from 1993 shows that there are more smokers in villages than in towns (2). Unlike Denmark, Greenland has experienced a decline in smokers’ average cigarette consumption (from 1993 to 2005) from 11.2 to 9.2 per day, whereas in Denmark there was an increase in the average cigarette consumption per capita until 2007, when a downward trend was seen (5). This may suggest that Greenlanders handle their knowledge about smoking differently than Danish smokers.

Since 1995, various smoking reduction measures have been implemented. There are heavy tax duties on tobacco and cigarettes. Smoking laws have restricted smoking in the workplace. There is a total ban on smoking in public buildings and in children's and educational institutions. There are health warnings printed in Danish and Greenlandic on all cigarette and tobacco packets. There is a ban on the sale of tobacco products to people under the age of 18, and a number of smoking cessation counsellors have been trained (4). On 1 October 2010, the smoking legislation was further tightened to prohibit smoking indoors in all public and private places such as restaurants, cafes, private clubs and associations.

## Aim

The aim of the study was to develop knowledge about how smoking forms an incorporated part of a social and cultural context in daily lives of unskilled residents of a small town in northern Greenland.

The study explored 2 main questions: (a) What significance do smokers attach to smoking in everyday life and (b) How do smokers integrate the public and private smoking rules into their smoking behavior.

## Material and method

An ethnographic method incorporating interviews, participant observation and informal conversation with an approach based on the understanding that knowledge is something that is created between people through observation and participation in everyday life (6, 7).

Participant observation along with informal conversation with nurses, a doctor at the hospital and random passers-by in the village where the first author met other smokers were important, since culture cannot easily be discussed directly and objectively by respondents who are living it and making their lives work on a practical level (6).

Field notes recorded the first author's movements in the small fishing village in northern Greenland in June 2010 for 5 weekdays, during which people's smoking habits were observed. A small village was included because more smokers live in villages than towns and cities. Unskilled workers with a maximum of 9 years of elementary school were chosen for interviews because they make up the largest group of smokers (2).

Four people were interviewed in depth. Two men and two women who met the following criteria: ethnic Greenlander (at least one grandparent is Greenlandic), aged 30–59 years, daily smokers and without chronic diseases attributable to smoking. Access to the smokers was facilitated by the local doctor, who identified potential participants.

The first author did the interviews, with 3 taking place in Danish and 1 in Greenlandic that was translated into Danish by a professional translator.

## Ethics

The project was conducted in accordance with the Declaration of Helsinki, Article II. Written information consent was obtained. The Research Ethics Committee for Scientific Research in Greenland approved the study.

## Analysis

A phenomenological hermeneutic interpretative approach was taken to data analysis, based on the approach described by Ricoeur (8) and further developed by Scandinavian researchers (9, 10).

Material from interviews and participant observations were transcribed and analysed. Ricoeur describes the interpretation of a text as an on-going dialectical movement between explanation and understanding. To understand a text is to follow its movement from significance to reference: from what it says to what it talks about. The interpretation method involved 3 analytical steps: naive reading, structural analysis and in-depth understanding (8). The naive reading aimed to be non-judgmental and to open up insight into the meaning of the text as a whole. A structural analysis was conducted in order to explain the text and to identify and formulate themes. As is apparent from Table I, this step took the form of a movement between units of meaning and units of significance in the text leading towards in-depth understanding.

**Table I:** Results of the structural analysis

Units of meaning (What is said)	Units of significance (What is being talked about)	Themes
“I smoke when I don't have anything else to do”	How smoking is part of daily life	Smoking behavior
“I get restless … don't know what I should be doing”	Physical and psychological feelings on giving up smoking	Withdrawal symptoms
“I would like to give up smoking, but I don't know how”	Lack of knowledge about help to stop smoking	Smoking cessation
“I've promised my children I'll give it up”	Bad conscience about continued smoking	Stigma

Critical interpretation aimed to develop a new understanding. The interpretation process drew out full sentences and meaning-laden expressions that relate to experiences of smoking, both from the interview text and the field notes from participant observations. These are interpreted and discussed below based on theory and other relevant research.

## Results

In a movement between units of meaning (what is said/quotations) and themes (what is being talked about), the structural analysis identified 4 themes: smoking behavior, withdrawal symptoms, smoking cessation and stigma.

In the following discussion, the 4 themes are presented separately for clarity (see Table I), but with the understanding that they interact with each other in reality.

## Smoking behavior

Smoking was described as a habit by all 4 participants. Three described smoking as something negative: “A bad habit in the morning, as soon as I come down, although I don't want to” and another informant, who generally smoked a pipe but smoked cigarettes when he drinks beer, said: “Unfortunately, I just do it, I'm a robot.” The wife of one informant, who was the only person at her workplace who smoked, stated: “I have a bad conscience. When I smoke at work, I wash my hands, brush my teeth, and chew gum.” In addition to it being a habit, for 3 of the 4 participants smoking was also associated with a bad conscience (i.e. feelings of guilt).

The participants smoked between 4 and 15 cigarettes a day and had been smoking for at least 20 years. Their spouses smoked too. The participants smoked more when they drank alcohol. “When I need beer, I start to smoke early in the day.” An informant who generally smoked a pipe, except for once or twice a month when he drinks alcohol and shares about 4 packs of cigarettes with friends, said: “If I stop smoking, I feel better within myself. I have strength of character, and can then stop drinking.” The informant indirectly indicated that he has a drinking problem.

The participants did not smoke so much when they were working, but rather when they relax or “are bored”. One Informant stated: “I smoke when I don't have anything to do, so I smoke a cigarette every 1 to 1.5 hours, mostly at the weekend.” Another informant said: “When I'm busy I don't smoke, it's mostly when I'm relaxed.” And a third stated: “I forget to smoke when the grandkids are here, when I think of smoking, I just go outside.”

The participants smoked less at work in part because it must fit into their work schedule. “I smoke when I've completed a big task and after breaks.” Another informant told us: “At work I smoke when others invite me out to smoke, but not every time … it has to fit in.”

Similarly, at home they smoked when they finished something. “I smoke after work, when I have eaten dinner, when I watch TV, and just before I go to bed.” Two of the four informants said that coffee and a cigarette go together.

## Smoking cessation

All the participants knew someone who did not smoke. Three had quit smoking for a period of time ranging from 3 months to a year. They all reported a need for smoking cessation. One informant had just been hospitalised with a heart condition. In the hospital, she was advised to give up smoking, but asked us “how do I stop?” and later added “but I don't have any guidance … there should be ads on radio and TV about how to quit.” Another interviewee said: “I think you can help me to give up smoking”. This interviewee also received advice while in the hospital to give up smoking, and responded positively to the idea.

One participant had attempted to quit smoking along with her partner for 3 months. It was during a period where her mother-in-law was seriously ill. They took up smoking again because they found that quitting smoking made them angry towards each other and other people. Two participants had also considered stopping smoking, as they were ill or had close relatives who were ill. One informant reduced her cigarette consumption from 15 to 10 cigarettes a day after a period of illness. She tried to buy nicotine gum to help her to stop, but she said it tasted nasty and she got severe headaches, so she only took 2 pieces The informant who does not want to give up smoking said: “I have tried to skip [the last cigarette] before I go to bed, but I can't.” Later she described why: “I cannot sleep when I don't have that last cigarette.” She would like to stop if her partner also stopped. The other participants also expressed that they would like to stop together with their partners, and they all expressed that they need help to quit.

## Withdrawal symptoms

When it comes to giving up smoking, it was the withdrawal symptoms that occupied all participants. One made the comment that: “We were shaking and needed a cigarette. Instead we should have gone for a walk … What prevents us giving up smoking is all the sulking … that period where you get mad at each other … we're afraid of all the negativity towards each other and towards others.” Another informant said: “When I stopped for a week, I got restless, did not know what to do, and when I drink coffee … I get angry as well … how do I stop? I need guidance.”

## Stigma

Many years prior to the study, all the informants had themselves implemented smoking restrictions in their own homes, and 3 interviewees have been in that situation for the last 15–17 years. Smoking restrictions had begun when they had children. Only one of the subjects never smoked inside, which meant less smoking in bad weather. The other 3 smoked in the living room, but if children or non-smokers were visiting they smoked in the kitchen under the extractor fan, behind a closed door, or even outside. One middle-aged, female Greenlander, who has lived in Denmark for many years, was on holiday and smoked on the street. She thought that the smoking restrictions are excessive: “It was cosier in the 1970s, when we smoked indoors.”

They were all characterised by experiencing guilt about smoking, and one was even bullied at work, where she was the only person who smoked. All participants said that the reason they smoked less at work was because they had something to do, and none mentioned that the reduction was due to workplace smoking restrictions.

## Discussion

The smokers expressed that they smoked the most when they were free from work and did not have anything else to do. Tulenius found that Danish smokers smoke more in “quiet times”, but when in the Danish context spoke of “quiet times” it was when interviewees gave themselves time during a stressful working day (11). The participants in this study did not talk about having had a stressful working day. Three of the 4 participants expressed that having a “good time” was essential for them. This was expressed by the fact that they wake up well before they go to work (between the hours of 4:30 and 6:30 am), that it was important that meetings occurred on time, and that the day started quietly. Warming and others wrote about this “good time” in a previous research project on Greenland values in child rearing, now expressed by a participant: “That his parents took it that you came in good time before you had to be there, as a good upbringing” (12). The value “good time” combined with the fact that each smoker smokes most when they have “good time,” may be a barrier for smoking cessation. In connection with having children, the participants had introduced smoking restrictions at home to prevent their children being exposed to the harmful effects of cigarette smoke. The participants have set up smoking restrictions in their own homes in the same time as the political systems starts to make restriction for smoking in public places, which can be seen as an acceptance of the political restriction. None of the responders question the political restrictions. It also seems that if close relatives are seriously ill, the participants consider giving up smoking; however, withdrawal symptoms present a barrier to cessation.

The value system surrounding human interaction in Greenland is based on placing great emphasis on the need to maintain harmony. The desire to quit smoking among informants from a small town can therefore be set in relation to the Greenlandic desire for harmony in daily life. Warming et al. examined the value system that underlies how children are brought up in Greenland. It points to a situation where:

Harmony is still a high priority in villages. A majority of those interviewed indicate that it will benecessary to downplay the desire and the need to always live in harmony. This was necessary in earlier societies, but in modern society, it would be desirable and necessary that young people learn to stand up and speak their minds, to come up with proposals to solve any social problems and to propose changes – even if these suggestions may be controversial and go against other people's perceptions. (12)

The fear of aggression is also described by Rønsager in Greenlanders’ health and illness beliefs spanning over the period 1800–1930:

Historically, traditional societies in Greenland have been small, meaning that unity among community residents was important for survival … The small community was therefore vulnerable to people who were different, those who could become aggressive, but also people who were mentally fragile. It was prudent to avoid conflicts, but this was not always possible. There were several ways to resolve conflicts, and common to them all was a low level of aggressiveness. (13)

Harmony value systems prevailed for many years as a key to survival in the small Greenlandic communities. The interviewees were all of an age that made it likely that they had grown up with a desire for harmony. This could be 1 reason why withdrawal symptoms occupied their minds so much. There has also been an international focus on withdrawal symptoms in relation to smoking cessation, including in a meta-analysis by Etter et al., who investigated the long-term effects of smoking cessation using nicotine replacement therapy (NRT). Those who received NRT were significantly twice (OR: 1.99) more likely to be smoke free than the control group receiving placebo (14). Three of the current participants wanted to stop smoking, had experienced smoking cessation, but failed in remaining abstinent. They asked for help to quit smoking. Etter et al. raised the question of whether smoking cessation should be looked at as a chronic disease instead of as an infectious disease with continued treatment (13).

The smokers interviewed were affected by pangs of conscience. One participant was bullied at work for being the only smoker. At the same time there seems to be ambivalence about smoking: the participants continued to smoke despite the fact that they had a bad conscience about being a smoker.

The interviewees all knew people who had stopped smoking or who had never smoked. Smoking could be considered as a normative behavior in Greenland since two-thirds of the population currently smoke (2). Three informants want to give up smoking, but this is hampered by aspects of their lifestyle such as seeking harmony in their community. The Danish philosopher, Juul Jensen, describes the power of different ways of life and society's moral responsibility: “You can become subject to the power of a way of life by taking its routines and patterns for granted” (15). The desire for harmony at all costs – when seen as a way of life with this kind of inherent power – persists in Greenland and hampers smokers’ attempts to quit smoking. From the perspective of Juul Jensen, it is society's responsibility that smokers are stuck in the old values of harmony, and based on society's duty to care for the smokers, one can say that society has a responsibility to help weak smokers to stop.

In Greenland, where two-thirds of the adult population are smokers, it seems that by virtue of smoking restrictions, society and non-smokers have created a message to smokers that the “norm” is to belong to the third of the population who do not smoke. Based on Goffmann's definition, one can say that smokers have become stigmatised:

Stigma is something experienced by an individual who, for one reason or another, is not able to achieve full social acceptance. The person is reduced in the collective consciousness from being an “ordinary” person to being an “impaired and undermined” person by being branded with – or assigned – a “stigma”. When a person is branded with a stigma, this simultaneously reaffirms “the others” normality. (16)

Another reason why smokers feel stigmatised could be that their knowledge about the harmful effects of smoking gives them a bad conscience about not having the strength of character to stop. In this way they can self-stigmatise themselves. This experience of stigma can be the underlying reason for their wish to give up smoking.

One of the informants tried to buy nicotine replacements, but the packaging indicated that it was too high a dose compared with her cigarette consumption, so she stopped using them because of this discrepancy. The first author checked out the supply of nicotine replacements in the local shop. It was noted that it was only possible to buy nicotine chewing gum, and then only in its strongest dose. One local medical secretary, who has been trained as a smoking cessation trainer, has not yet set up any cessation courses, and the local council's prevention consultant focuses mainly on young people who are sniffing glue and on the prevention of suicide.

In summary, there is a desire to stop smoking, but withdrawal symptoms form a barrier, and help to stop smoking is requested but is not easily available.

## Conclusion

The study illustrates that smokers have a lot of time on their own when they are free from work, and it is then that they are most likely to smoke. A disjuncture can also be seen between the desire to quit smoking and the knowledge, will, and strength required. The social and cultural context also impacts the low degree of smoking cessation. Official smoking restrictions have led to non-smokers being regarded as the “norm,” despite the fact that the majority of the adult population smokes. This implies that there is a process of self-stigmatisation of smokers.

The emphasis on achieving and maintaining harmony in relation to those around you seems to be crucial. It appears to create a barrier that prevents smokers giving up smoking when they experience withdrawal symptoms that threaten the harmonious atmosphere. Withdrawal symptoms and lack of knowledge about how to stop are barriers to reaching their goal.

## Perspectives

One action which could increase the number of non-smokers would be the establishment of smoking cessation services adapted to the Greenlandic culture, taking into account the values of harmony and having good time. Smokers would be given guidance on, for example what can be used instead of cigarettes and where knowledge and medical treatment of withdrawal symptoms are readily available.
